# Rationales of Cold Plasma Jet Therapy in Skin Cancer

**DOI:** 10.1111/exd.70063

**Published:** 2025-02-19

**Authors:** Sander Bekeschus, Debora Singer, Gishan Ratnayake, Klaus Ruhnau, Kostya Ostrikov, Erik W. Thompson

**Affiliations:** ^1^ Department of Dermatology and Venerology Rostock University Medical Center Rostock Germany; ^2^ ZIK Plasmatis Leibniz Institute for Plasma Science and Technology (INP) Greifswald Germany; ^3^ Department of Radiation Oncology Princess Alexandra Hospital Brisbane Queensland Australia; ^4^ neoplas MED Greifswald Germany; ^5^ School of Chemistry and Physics and Centre for Biomedical Technologies Queensland University of Technology Brisbane Queensland Australia; ^6^ School of Biomedical Sciences and Centre for Genomics and Personalised Health Queensland University of Technology Brisbane Queensland Australia; ^7^ Translational Research Institute Brisbane Queensland Australia

**Keywords:** actinic keratosis, AK, basal cell carcinoma, BCC, combination therapies, malignant melanoma, SCC, squamous cell carcinoma, tumour wound

## Abstract

Skin cancer affects millions of patients worldwide, and its incidence is increasing. Current therapies targeting skin tumour subtypes, such as basal cell carcinoma, cutaneous squamous cell carcinoma, melanoma and actinic keratosis, vary in their degree of effectiveness and tolerability, motivating new research avenues on complementing treatment strategies. Cold medical gas plasma is a partially ionised gas operated at about body temperature and generates various reactive oxygen and nitrogen species simultaneously. A range of medical gas plasma devices has proven safe in thousands of patients and is an approved medical product for dermatology conditions, such as nonhealing wounds, in Europe and, more broadly, for clinical trials. Extending potential gas plasma applications in the field of dermato‐oncology is therefore plausible, especially in light of the strong preclinical evidence and early clinical data. This review summarises existing work on gas plasma treatment, focusing on approved jet plasmas in skin cancer and outlining central mechanisms and treatment concepts. It also provides a concrete perspective on integrating medical gas plasma treatment into existing skin cancer therapy schemes, encouraging translational scientists and clinicians to enable gas plasma‐assisted cancer care through clinical research.

## 1 Introduction

Skin cancer affects millions of patients worldwide, and its incidence is increasing [1, 2]. The disease is classified into several subtypes. The most frequent is basal cell carcinoma (BCC). At late diagnosis, BCCs have a strongly invasive phenotype but rarely metastasize to distant body parts. Cumulative exposure to UV light throughout life strongly correlates with disease onset, but BCC lesions can also occur in areas of the body exposed to less sunlight. Sunlight exposure is also a major driver for the second‐most frequent type of skin cancer, cutaneous squamous cell carcinoma (cSCC). In many cases, cSCCs develop from precancerous malignant lesions referred to as actinic keratosis (AK), *carcinoma* in situ. AKs are dry, scaly patches, usually at body locations frequently exposed to sunlight. AKs are not classified as skin cancer per se due to their lack of invasiveness, but they occur often. For example, about 50% of people over 60 years old are thought to have at least one AK lesion, making AK treatments a significant issue in the healthcare systems across the globe. About 1%–10% of AK lesions can develop into SCC, but they do not metastasize [3]. Both BCC and SCC also frequently form superficial, nonhealing ulcerative lesions that are difficult to treat. Melanoma is another skin cancer that develops from the skin's melanocytes. It is known for its aggressive metastases formation, especially in the brain and lung, and is the cancer with the highest mutation rate in men [4]. There are also less common types of skin cancer, such as Merkel cell carcinoma, cutaneous lymphoma and various sarcomas. Throughout the past decades, scientific advances have enabled earlier detection and led to the discovery of several types of targeted therapies, including immunotherapies, which have significantly reduced skin cancer‐related mortality over recent years [5]. Despite this positive trend, skin cancer is still fatal in many patients, especially those with highly progressed disease and acquired or inherent therapy resistance. In addition, skin cancer treatment modalities can be suboptimal in terms of tumour growth control and side effects. This motivates and justifies further research into identifying new and complementary therapeutic avenues to benefit current and future skin cancer patients.

In the last decade, a novel medical technology based on cold gas plasma exposure has entered medical practice. In 2013, the first gas plasma device received clinical evidence‐based approval to treat acute and chronic wounds, ulcers and pathogen‐related skin diseases [6]. There are different major principles in the electrical design and operation modes of gas plasmas, such as plasma jets operated with external feed gas [7] and dielectric barrier discharges (DBDs) that can also be operated in the air [8]. Notwithstanding, all gas plasmas are multicomponent systems that include electric fields, electrons and ions, a broad spectrum of light (UV, visible and NIR), moderate thermal radiation and reactive oxygen and nitrogen species (abbreviated to only ROS here because RNS also carry oxygen) generation [9].

**FIGURE 1:**
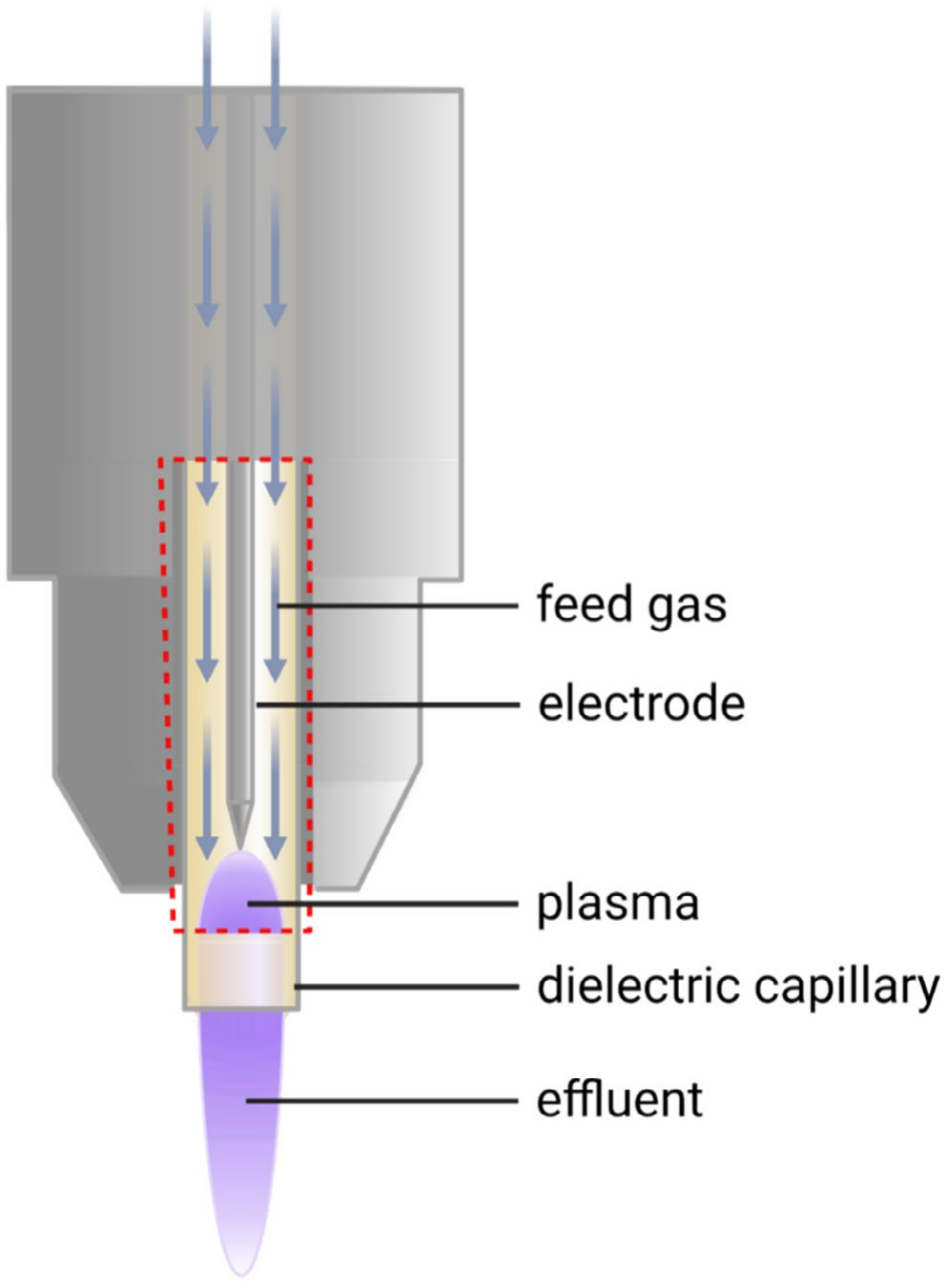
The principle of gas plasma generation based on the plasma jet principle.

For medical applications, two crucial criteria of medical gas plasma systems are their operation at temperatures that are close to body temperature and acceptable patient‐leakage currents, as outlined by first attempts at medical gas plasma source standardisation in Germany based on an industrial pre‐norm [10]. Independent of the gas plasma device utilised, many congruent effects have been observed in skin cancer research. What differs is usually the efficacy of tumour cell killing; for example, some devices may achieve sound anticancer effects in a few dozen seconds of treatment time, while others require several minutes or more. The uniformity of results across different treatment times is explained by the fact that ROS are a primary driver of gas plasma‐mediated biomedical effects [11]. At the same time, the composition and reactivity of the individual types of ROS in the reactive species mixture also affect the extent of anticancer effects, as was demonstrated in, for example, lung cancer [12], leukaemia [13] and melanoma [14] cells, illustrating the complexity of the topic of defining the optimal gas plasma cancer treatment modalities and regimens. Therefore, discussing potential similarities and differences between different gas plasma devices, electrical configurations, gas feed compositions and rates, treatment spots, durations and distances, to mention only a few parameters [15], is not only out of the scope of this review but also not possible due to the lack of head‐to‐head comparison studies between, for example, different devices (with a few exceptions [16, 17]). In addition, we will not discuss indirect treatments based on gas plasma‐oxidised liquid (also referred to as plasma‐activated medium (PAM), plasma‐treated liquid (PTL), plasma‐conditioned media (PCM), plasma‐activated water (PAW), plasma‐activated Ringer's lactate (PAL) and some others) because its primary mode of action is based on biochemical effects of reactive species produced through the plasma pretreatment of these media. Specifically, several studies provided evidence that the effects observed with this indirect approach using plasma‐treated liquids can be abrogated using hydrogen peroxide (H_2_O_2_‐)scavenging enzyme catalase or phenocopied by adding H_2_O_2_ alone [18, 19, 20]. With direct gas plasma treatments, much broader ROS patterns can be produced in a spatio‐temporally restricted manner, and promising preclinical results have been achieved in the past decades for skin cancer treatment. Especially, plasma jets were used in preclinical investigations on different skin cancer models. Typically, plasma jets consist of a pin electrode located in a dielectric tube and a grounded outer electrode (Figure 1). A feed gas (e.g., a noble gas) is led through the dielectric tube, creating the gas plasma at the tip of the device when an electrical field is applied [21]. Although plasma jet devices need equipment such as gas cylinders to provide the feed gas, making them less portable than the DBD devices, they offer a higher flexibility in their utilisation. With such jet devices, an exact application of plasmas to only a few millimetres of the skin surface is possible, enabling the treatment of small lesions while leaving out the surrounding healthy tissue. At the same time, the length of a plasma jet effluent allows for reaching deeper cavities, as one would find in the area of resected tumours or generally in varying surface morphology. By multiplying the number of jets (multijet plasma devices) [22], the possibility of treating larger surfaces as needed in cases of field cancerisation is also given.

## 2 Preclinical Studies in Gas Plasma Skin Cancer Treatment

Several major skin cancer types have been investigated following gas plasma exposure within experimental models. Effects of the plasma jet device kINPen, including its clinically certified version, were investigated in multiple preclinical studies successfully against skin cancer in experimental setups in vitro and in vivo (Table 1), and broadly similar findings were obtained using noncertified plasma sources (Table 2).

**TABLE 1:** Preclinical studies on gas plasma skin cancer treatment using the kINPen plasma jet.

kINPen: Gas type, max. exposure	In vitro	In vivo	Ex vivo (human)	Main findings	Ref.
				Malignant melanoma	
Argon/helium ± oxygen, 4 min	B16F10, SK‐Mel‐28	B16F10 (syngeneic)	—	Melanoma inactivation was dependent on feed gas composition; gas plasma‐inactivated melanoma cells given as protective vaccine were immunogenic; gas plasma‐induced immunogenic cell death (ICD); combination effect of gas plasma and imiquimod	[32]
Argon, 5 min	—	B16F10 (syngeneic)	—	Gas plasma treatment delayed melanoma tumour growth; combination of electrochemotherapy and gas plasma prolonged animal survival	[46]
Argon, 1 min	A375	—	—	Combination effects of gas plasma and novels mall molecules in vitro, affecting several signalling pathways	[120]
Argon, 2 min	B16F10	—	—	Increased toxicity and immunogenicity, including MHC class I and CRT expression, following gas plasma exposure	[34]
Argon, 2 min	SK‐Mel‐28, MeWo, Ma‐Mel‐86a, 501‐Mel, A375	—	Malignant melanoma Stage IV	Tumour cells showed different sensitivities towards gas plasma treatment; basal xCT expression was associated with gas plasma treatment resistance	[23]
Argon, 2 min	501‐Mel, A375, Ma‐Mel‐86a, MeWo, SK‐Mel‐19, SK‐Mel‐28, SK‐Mel‐29, SK‐Mel‐63, SK‐Mel‐147	—	—	Gas plasma sensitivity varied greatly throughout different cancer cell lines; association of gas plasma resistance with high cholesterol levels and strong correlation with tumour cell metabolism	[39]
Argon, 30 s	B16, SK‐Mel‐28	—	—	Enhanced cytotoxicity of doxorubicin via gas plasma‐induced upregulation of SLC22A16 transporter in melanoma cells	[45]
Argon, 30 s – 5 min	SK‐Mel‐28, MaMel‐86a, B16F10	B16F10 (syngeneic)	—	Synergistic effects of gas plasma combined with mitochondria‐targeted drugs; immunomodulatory effects and gas plasma‐induced immunogenic cell death (ICD) in melanoma cells; combination effects of gas plasma and anti‐PD1	[35]
Argon, 90 s	A375	—	—	Gas plasma treatment increased surface marker expression and proinflammatory mediator production; increased toxicity in NK‐cell co‐cultures	[61]
Argon, 5 min	—	A375 (xenograft)	—	Reduction of tumour cellularity and reduced tumour growth; detection of gas plasma‐induced ROS formation in the tumours	[64]
Argon, 60 s	SK‐Mel‐28, B16F0, B16F10	—	—	Increased cytotoxicity of nanoparticles combined with gas plasma treatment	[121]
Argon, 15 s	B16F10	—	—	Additive toxicity of gas plasma combined with radiotherapy	[122]
Argon, 2 min	—	—	Malignant melanoma Stage IV	Gas plasma treatment induced apoptosis; immunomodulatory effects	[123]
Argon ± various shielding, 89 s	MNT‐1, SK‐Mel‐28 (spheroids)	—	—	The percentage of oxygen and nitrogen in the immediate vicinity of the argon plasma has negligible effects on anti‐melanoma toxicity and does not promote melanoma cell metastasis	[124]
Argon ± nitrogen/oxygen/humidity, 120 s	B10F10	—	—	Argon plasma feed gas variation changed the plasma ROS chemistry and dramatically affected melanoma growth and immunogenicity (CRT expression)	[14]
Argon, 30 s	B16F10	—	—	Gas plasma‐induced cytotoxicity; immunomodulation and immune cell activation	[125]
				Squamous cell carcinoma (SCC)	
Argon, 3 min	HNO97	—	Oral SCC (maxilla‐facial region)	Increased apoptosis induction by gas plasma in SCC cell lines and ex vivo–treated patient tissue compared to healthy tissue	[65]
Argon, 90 s	A431	—	—	Gas plasma treatment modulated surface marker and proinflammatory mediator expression in SCC cells towards augmentation of NK‐cell activity	[61]
Argon, 5 min	—	A431 (xenograft)	—	Gas plasma treatment significantly reduced tumour growth in vivo but did not induce apoptosis	[64]
Argon, 2 min	A431, SCC‐13	—	cSCC tumour	Gas plasma‐induced cell death; apoptosis induction; decreased CCL5, GM‐CSF and PDGF‐AA levels	[50]
Argon, 5 min	SCC‐4, SCC‐9	—	Oral SCC tumour	Dose dependency of gas plasma treatment; apoptosis induction; no genotoxic effects	[126]
Argon, 1 min	A431, SCC‐7 (spheroids, *in ovo*)	A431 (xenograft)	—	Combination effects of gas plasma and small molecules in 3D tumour spheroid culture, *in ovo* and in vivo	[120]
Argon, 2 min	A431	—	—	Gas plasma‐induced cytotoxicity and apoptosis; cell density‐dependence; reduced migration; combination effects with an indirubin‐derived small molecule	[127]
Argon, 5 min	A431, SCC‐25	A431 (xenograft)	—	Adaptation processes following chronic low‐dose gas plasma exposure correlated with IL1R2 expression; reduced tumour growth in vivo	[128]
				Basal cell carcinoma (BCC)	
Argon, 2 min	—	—	BCC tumour	Gas plasma is cytotoxic via apoptosis in BCC cells; immunomodulatory effects of gas plasma	[50]
				Actinic keratosis (AK)	
Argon, 3 min	—	UVB‐induced lesions	—	Gas plasma treatment controlled growth of UVB‐induced lesions; local modulation of redox balance; reduced proliferation of malignant keratinocytes associated with reduction of Nrf2 expression	[49]

**TABLE 2:** Preclinical studies on gas plasma skin cancer treatment using other noncertified plasma sources.

Plasma source: Gas type, max. exposure	In vitro	In vivo	Ex vivo (hu.)	Main findings	Ref.
				Malignant melanoma	
μ‐DBD, air, 5 min	G‐361	G‐361 (xenog.)	—	Combination of gas plasma and silymarin nanoemulsion increased cytotoxicity and minimised malignancy markers in melanoma cells	[47]
Jet, argon, 45 s	B16F10	B16F10 (syng.)	—	Inactivation of melanoma tumour cells by gas plasma; combination effects of gas plasma and dacarbazine	[36]
miniFlatPlaSter, air, 2 min	Mel‐Im, Mel‐Juso	—	—	Dose‐dependent antitumor effects ranging from senescence induction to strong apoptosis induction	[24]
Jet, neon/argon, 90 s	B16	B16 (syng.)	—	Melanoma cells showed sensitivity towards gas plasma treatment	[25]
DBD, air, 3 min	B16, A375	—	—	Combination effects of gas plasma and chemotherapeutics (bleomycin, paclitaxel and dacarbazine)	[41]
Jet, argon, 90 s	B16	B16 (syng.)		Gas plasma mediated antitumor effects by increased autophagy and apoptosis; combination effects of gas plasma and starvation	29
DBD, argon, 30 s	—	RET‐transgenic mice (syng.)	—	Single gas plasma treatment modulated tumour growth and invasion regulators in melanoma without BRAF mutation	[26]
Jet, helium, 30 s	Mel‐RM, Mel‐007, Mel‐JD	—	—	Gas plasma‐induced apoptosis in melanoma cells is mediated by ASK1 activation via TNF signalling	[28]
Jet, 90 s	Hs 895.T	—	—	Higher intracellular ROS production in melanoma than in nonmelanoma counterparts; melanoma cells showed greater sensitivity towards gas plasma‐induced ROS	[27]
DBD, air, 10 s	B16F10, A375	B16F10 (syng.)	—	Gas plasma‐induced immunogenic cell death (ICD) in melanoma cells; ICD depends on short‐lived ROS generated by gas plasma	[33]
Jet, helium, 40 s	B16F10, SK‐Mel‐2	—	—	Combination with gas plasma improved apoptotic effects of doxorubicin and liposomal doxorubicin and reduced aggressiveness and metastatic potential of melanoma cells	[42]
Jet, argon, 45 s	B16	B16 (syng.)	—	Gas plasma damaged melanoma cells over nonmalignant cells; effective reduction of melanoma tumour size	[37]
Jet, helium, 6 min	B16F10	B16F10 (syng.)	—	Direct gas plasma treatment outperformed exposure to gas plasma‐treated medium regarding cell death and tumour growth reduction	[43]
miniFlatPlaSter, air, 2 min	Mel‐Juso, Mel‐Im	—	—	Gas plasma exposure induced acidification mediating anticancer effects; gas plasma affected intracellular Ca^2+^ levels of melanoma cells	[30]
μ‐Jet, helium ± oxygen/nitrogen, 11 min	Malme‐3 M, SK‐Mel‐28	—	—	Gas plasma effects depended on feed gas composition, gas flow, treatment time, cell type and treatment method	[40]
Jet, helium/oxygen, 4 min	A375	—	—	Synergistic effect of continuous doxorubicin treatment with subsequent gas plasma treatment	[44]
miniFlatPlaSter, air, 2 min	Mel‐Im, Mel‐Juso	—	—	Gas plasma generated acidified nitrite, mediating strong inhibition of melanoma cells	[31]
Torch, helium/air, 10 s	WM793B, 1205Lu	—	—	Gas plasma‐induced apoptotic cell death in melanoma cells grown in 2D and 3D	[38]
				Squamous cell carcinoma (SCC)	
Jet, helium, 45 s	JHU‐022, JHU‐028, JHU‐029, SCC25	—	—	Dose‐dependent and killing of head and neck SCC cells by gas plasma	[57]
Torch, helium/oxygen, 1 s	FaDu, SNU1041, SNU899	FaDu (xenog.)	—	Gas plasma‐induced apoptosis in head and neck SCC cells and inhibited tumour growth, mediated by MAPK‐dependent mitochondrial ROS	[62]
Jet, air/nitrogen, 4 min	MSKQLL1, SCC1483	—	—	Gas plasma treatment inhibited migration and invasion of SCC cells by inhibiting focal adhesion kinase and matrix metalloproteinase activity	[55]
Torch, helium/oxygen, 1 s	MSKQLL1, SCC1483, SCC15, SCC25	—	—	Gas plasma‐induced apoptosis is mediated via DNA damage and ATM/p53 pathway‐triggered cell cycle arrest	[129]
Torch, helium/oxygen, 1 s	MSKQLL1, SCCQLL1, HN6, SCC25, SCC15, Cal27, SCC1483	—	—	Gas plasma combined with cetuximab reduced the invasiveness of cetuximab‐resistant SCC cells	[59]
Torch, helium/oxygen, 1 s	SCC15, FaDu, SCCQLL1, SCC1483, SNU1041, SCC7, HN6	SCC7, SCC15 (xenog.)	—	AKT degradation via MUL1 expression induced cell death in gas plasma‐treated SCC cells	[63]
Jet, argon, 2 min	SAS, Ca9‐22, HSC‐2, HSC‐3, HSC‐4, Sa3, Ho‐1‐u‐1	—	—	Gas plasma‐induced cell death in SCC was associated with ferroptosis, reduction of migration, invasiveness and colony formation	[56]
Jet, argon, 3 min	SCC‐15	—	—	Combination effects of gas plasma and cisplatin in SCC	[60]
				Basal cell carcinoma (BCC)	
Jet, argon, 60 s	BCC	—	—	Short‐lived species in gas plasma‐induced apoptosis in BCC cells	[67]
				Actinic keratosis (AK)	
—	—	—	—	—	—

### 2.1 Malignant Melanoma

Most studies have been conducted using murine and human melanoma cells. These range from in vitro cell line testing from 2D to 3D cell line models to animal studies, mostly in mice. Only one study involved human melanoma skin cancer tissue from punch biopsies [23]. Effective inhibition of melanoma cells was shown in vitro and in vivo [24, 25, 26, 27], and induction of apoptosis and autophagy, acidification and altered Ca^2+^ homeostasis were identified as contributors to the modes of action in gas plasma cytotoxicity [28, 29, 30, 31]. Additionally, some studies were able to show an immunogenic effect of the gas plasma treatment on melanoma cells [14, 32, 33, 34, 35]. Although a selective inhibition of melanoma cells was suggested in some studies [36, 37, 38], no study compared gas plasma cytotoxicity in melanocytes, and no other nonrelated noncancer cells to that observed in melanoma cells. In addition, differences in gas plasma sensitivities were observed when different cell types and/or plasma settings were compared [23, 39, 40]. Importantly, in combination treatments—especially with chemotherapeutics—gas plasma was able to improve anti‐melanoma effects significantly when used together [41, 42, 43, 44, 45, 46, 47], offering the possibility to reduce dose‐dependent side effects of such conventional treatments or to target melanoma more effectively.

### 2.2 Squamous Cell Carcinoma

For cutaneous SCC (cSCC), only a few articles are available [48, 49, 50]. At the same time, there is a considerable number of articles on oral SCC, which can ulcerate visibly on the head and neck of cancer patients, so it is accessible to surface treatments, including cold medical gas plasmas [51]. There are differences but also similarities between cutaneous and oral SCC based on histopathological [52] and genetic [53, 54] features, so both entities are listed in the context of gas plasma therapy. In vitro studies revealed the positive effects of gas plasmas on reducing SCC cell migration and invasiveness [55, 56], inducing cytotoxic effects [57, 58] and improving chemotherapy efficiency [59, 60]. In one study using the A431 cSCC line, a probable immunomodulatory effect of the gas plasma was observed [61], as already mentioned for malignant melanoma. Furthermore, a few studies on SCC gas plasma treatment were conducted in vivo [62, 63, 64], and one performed ex vivo treatment of SCC tissue biopsied from patients [65], all showing a reduction of growth or apoptosis induction in the SCC tumours or tissues.

### 2.3 Basal Cell Carcinoma

Although it is the most frequent skin cancer entity, research lacks appropriate BCC cell lines [66], impeding in vitro studies. Up to now, only two studies have investigated the effects of gas plasma exposure in basal cell carcinoma, which either used a self‐established BCC cell line [67] or ex vivo‐treated punch‐biopsied BCC tissue from patients [50]. Both studies were able to show apoptosis induction after the gas plasma treatment, and again, there was evidence of immunomodulatory effects.

### 2.4 Actinic Keratosis

Actinic keratosis is challenging to model in vitro because frequent UV exposure of, for example, human keratinocytes is frequently associated with cellular senescence [68] via p38 signalling [69]. However, precancerous AK‐like lesions up to invasive cSCCs can be induced in vivo upon frequent UV exposure of mice, such as immunocompetent hairless SKH‐1 mice [70]. In one study, such a model was employed to unravel the protective effects of gas plasma exposure against UV‐induced lesions [49].

### 2.5 Clinical Evidence

Despite the various promising results found in in vitro, in vivo (mouse) and ex vivo (human) treatment of skin cancers with plasma, the clinical study situation is limited (Table 3). Although broad evidence was gained from preclinical studies on malignant melanoma, so far no case series or clinical studies have been conducted investigating the efficacy of plasma in melanoma. The lack of preclinical studies on BCC is visible in the absence of clinical studies on this nonmelanoma skin cancer (NMSC) type. For SCCs, only the oral form with ulcerations on the head and neck region was tested in palliative treatment settings. Notably, an absence of severe side effects of the plasma treatment [71] up to reduced pain [72] was detected. Furthermore, an induction of apoptosis in the plasma‐treated tissue could be proved [73]. The most extensive case series and clinical studies were conducted on actinic keratosis. Common to all was the absence of adverse effects upon plasma treatment. Summarised, these studies showed improvement of AK lesion features, reduction in the lesion count up to complete reversal of the AK and skin rejuvenating effects [74, 75, 76, 77, 78]. The outcome of these studies paves the way for further clinical trials on a larger scale and should encourage the expansion of studies to other skin cancer types like malignant melanoma and cSCC.

**TABLE 3:** Clinical studies and case series on gas plasma skin cancer treatment.

Skin cancer type	Plasma source	# Patients	Combination	Main findings	Ref.
Actinic keratosis	Adtec SteriPlas	12	—	Gas plasma improved chronic photodamage features; no skin atrophy‐induced	[74]
Actinic keratosis	Maximum electrosurgery unit with maximum beamer	1	—	Gas plasma cured recalcitrant AK after single treatment; no relapse after 26 months	[75]
Actinic keratosis	Custom made	5	—	Significant improvements up to complete reverse of AK after single gas plasma treatment; no adverse effects	[76]
Actinic keratosis	Adtec SteriPlas	60	—	Gas plasma reduced AK lesions as effective as diclofenac treatment; no adverse side effects compared to diclofenac; rejuvenating of skin was observed	[77]
Actinic keratosis	Adtec SteriPlas	7	—	Downgrading of Olsen Score and reduction of AK lesion count after gas plasma treatment in field cancerisation; no adverse effects; skin rejuvenating effects	[78]
Cutaneous squamous Cell carcinoma	—	—	—	—	—
Oral squamous Cell carcinoma	kINPen MED	6	—	Reduced odour (contamination) and pain medication requirements after gas plasma treatment in advanced oral SCC	[72]
Oral squamous Cell carcinoma	kINPen MED	2	—	Gas plasma‐mediated effects on microcirculation in advanced oral SCC and postoperative tumour wounds	[130]
Oral squamous Cell carcinoma	kINPen MED	20	—	Gas plasma treatment lacks severe side effects in palliative oral SCC treatment	[71]
Oral squamous Cell carcinoma	kINPen MED	21	—	Gas plasma treatment induces apoptotic cell killing and tumour surface responses (vascular stimulation or contraction of ulceration rims) in advanced oral SCC	[73]
Basal cell carcinoma	—	—	—	—	—
Malignant melanoma	—	—	—	—	—

## 3 Main Medical Gas Plasma Application Concepts for Skin Cancer

**FIGURE 2:**
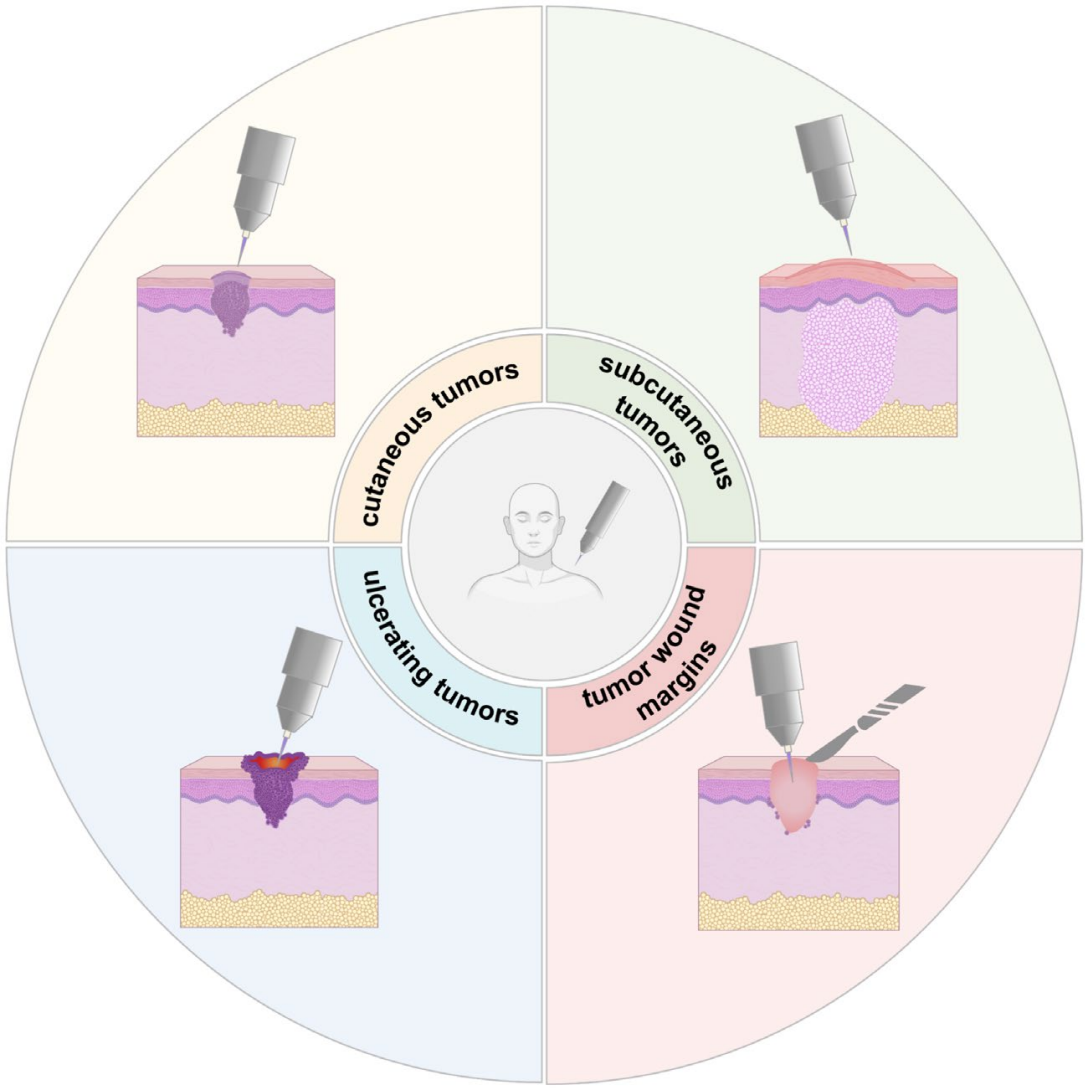
Clinical concepts of using medical gas plasma in skin cancer therapy.

As mentioned above, the clinically most relevant skin cancer entities are malignant melanoma, cSCCs, BCCs and—as carcinoma in situ and SCC precursor—AKs. There are also others, such as Merkel cell carcinoma and skin lymphoma, that are not covered in this review. To date, no country or region has approved gas plasma technology for any type of skin cancer or topical ulcerating cancer, such as oral squamous cell carcinoma (head and neck cancer) and some breast cancers. However, given the easy accessibility of cancers, on or in the skin, there are ample opportunities (Figure 2) to unravel the potential benefits of using gas plasma technology alone or in combination with established oncology treatment modalities.

### 3.1 Cancers Ulcerating Through the Skin

One option is the direct treatment of skin cancer cells visible on the surface. This is the case for ulcerating cancer lesions where tumour cells can be directly exposed to the gas plasma treatment. This scenario allows repeated gas plasma treatment sessions over weeks to months, as was implemented in a cohort of patients suffering from large ulcerating head and neck cancer regions, as antimicrobial palliative treatment [79]. Furthermore, this approach allows clinicians to gain experience on which tumour region demands longer or shorter gas plasma exposures, depending on the tissue responses observed. In such a setting, gas plasma treatment would also allow the control of infections and biofilms, and co‐morbidities frequently observed in ulcerating tumour tissues.

### 3.2 Intradermal Skin Cancer

A second option would be the gas plasma treatment of skin cancer cells located intradermally or even partially within the stratum corneum. This is the case for some types of cSCCs and BCCs, and it is always the case for AKs. In these cases, gas plasma treatment effects would need to penetrate across dead skin barriers of minor or major thickness, mainly composed of faded keratinocytes, extracellular matrix (keratin squames) and lipids. This scenario also allows repeated and frequent exposure of the skin cancer lesions to the gas plasma at desired plasma exposure durations and repetition frequencies. However, the degree to which the reactive agents (e.g., ROS) produced by the gas plasmas can penetrate tissues is unclear. The extent of penetration may depend on the operating gas, the plasma exposure time and perhaps also the specific ROS types generated, as some types may reach further into the skin than others. In general, it should be assumed that the majority of gas plasma‐derived ROS react with biomolecules nearby first, thus producing a gradient of ROS concentration from the gas plasma source in contact with the tissue into that tissue that decays linearly or even logarithmically [80, 81]. Even in the absence of biomolecules, the lifetimes of most of the gas plasma‐generated ROS are below a few seconds. Possibly, electric fields could also contribute to deep‐tissue gas plasma effects, but reliable evidence of this effect is scarce. In mice receiving repeated UV dosages to actinic keratosis and cSCC‐like lesions, repeated gas plasma exposure was shown to reduce lesional growth [49]. However, the thickness of murine skin is only 10% of that of humans.

### 3.3 Subcutaneous Cancer Lesions

A third possibility of the gas plasma skin cancer treatment would be for subcutaneous lesions. In this scenario, an intratumoral localisation of gas plasma‐derived effectors in the tumour tissue is even more unlikely than in the case of intradermal cancer, mainly because of the thickness of human skin. To our knowledge, there is only one pilot attempt at direct malignant melanoma metastasis, which can occur subcutaneously or intradermally in a patient, resulting in skin whitening but no clinical response to repeated gas plasma treatment [82]. These considerations are in stark contrast to preclinical work, as many dozens of subcutaneously injected tumours of different types have been successfully reduced using repeated and direct gas plasma exposure (Table 1 and Table 2). The 10‐fold difference in skin thickness and composition to human skin, however, hampers the translational value of these findings, and experimentation in animals with skin more similar to humans (e.g., live pigs) should be undertaken.

### 3.4 Surgical Tumour Bed Gas Plasma Exposure

A fourth approach to direct plasma exposure in skin cancer is the treatment of surgical margins following tumour excision. It is known that tumour micrometastasis can spread into the surrounding tissue, being macroscopically undetected by the surgeon and microscopically undetected by the pathologist who can only evaluate a few 5‐ to 10‐μm‐thick sections of the tumour, but not all tumour‐to‐healthy‐tissue border regions, for R0 or R1 staging. Positive surgical margins (i.e., cancer cells at or close to the edge of the resected tissue) are a key indicator of local recurrence in most cancer types, including skin cancers, with pathologically positive or close surgical margins leading to subsequent resection to ensure maximal removal of residual cancer cells. This is despite local control with adjuvant radiotherapy in many cancer types (is it common in SCC management?). For invasive cSCC, it is recommended to have a 5 mm clear margin in low‐risk cases and 6–10 mm in high‐risk cases [83]. That, combined with radiotherapy, has kept the local recurrence rates down to less than 1%. Nonetheless, in striving to eradicate the local recurrence of the surgical region, cold gas plasma treatment of the surgical bed could be tested. The Canady Cold Helios Plasma device has been designed specifically to spray gas plasma along the surgical margins to eradicate residual cancer cells after tumour resection. This device is being tested in a Phase I clinical trial, with partially promising results so far [84]. This method may be used to kill residual cancer cells. It could have the added benefit of promoting immunological clearance, both locally for subadjacent cells and potentially also systemically since gas plasma exposure has been proposed to stimulate immune recognition of malignant cells [85, 86, 87]. Ultimately, showing that gas plasma treatment improves local recurrence rates relative to surgery only and/or allows reduced radiation treatment will be essential. Experimentally, this reduction in regrowth after surgery has been demonstrated successfully in mice for melanoma and breast cancer [88]. As an added benefit, the gas plasma exposure of tumour wounds could reduce wound infection occurring postoperatively.

### 3.5 Gas Plasma Technology as an Enabling Anticancer Modality

There are more concepts of how gas plasma could be included in oncology treatment schemes. For instance, tumour material, such as tumour biopsies, could be used to prepare anticancer vaccines whose immune‐stimulating effects could be enabled or promoted by the gas plasma treatment. This concept, including various aspects of the gas plasma‐mediated immune stimulation, has recently been reviewed [87]. Briefly, gas plasma treatment of tumour cells has been shown to stimulate their immune recognition, which underpins the potential use of gas plasma‐treated tumour cells as personalised tumour vaccines. This effect has been demonstrated in vitro and in vivo melanoma systems [32, 33]. It holds great promise as it can easily be adapted to modify excised tumours with enhanced vaccination potential and little risk or adverse outcomes. Another option is the utilisation of hydrogels for cancer treatment, for example, by filling cancer wound resection margins with gas plasma‐treated hydrogels, as recently reviewed [89]. This concept allows for controlled release of ROS over a 1‐ to 2‐week period, and the main question here is how the hydrogel can be produced reproducibly and evenly. In addition, it remains to be investigated in the future if gas plasma treatment concepts are economically competitive compared to the production of ROS‐loaded hydrogels with long‐lived oxidants generated by traditional chemical means. In general, the ROS chemistry in gas plasma‐treated hydrogels is subject to ongoing investigations, including efforts to enhance the retention of shorter‐lived ROS species [81, 90, 91] and enhance immunomodulation for improved immunotherapy [92]. A third gas plasma‐enabled concept is the use of gas plasma‐treated liquids (also referred to as gas plasma‐activated medium (PAM), plasma‐activated liquid (PAL), plasma‐conditioned medium (PCM), gas plasma‐oxidised liquids (POS) and many other names). However, it is commonly acknowledged that most of these liquids, especially at physiological pH and with clinical relevance, such as sodium chloride (NaCl), mostly contain long‐lived ROS, that is, hydrogen peroxide, nitrite and nitrate [18, 19, 20], making plasma processes for their production challenging in terms of standardisation. Operationally, they may also be less controllable than adding such single species from production with pharmaceutical quality. At the same time, plasma processes may have a better environmental footprint if directly produced at, for example, solar power plants within residual‐free production processes.

## 4 Integration of Gas Plasma in Existing Skin Cancer Treatment Schemes

When used alongside existing definitive and adjuvant therapies, gas plasma could play a potentially significant role in managing both early and advanced skin cancers. In evaluating the clinical application of gas plasma for skin cancer, an important consideration is the limited penetrative abilities of the reactive species into the skin tissue [93]. Thus, the gas plasma must either be in direct contact with tumour cells on the skin surface or be used to treat a thin layer underneath because the plasma‐generated reactive agents can only permeate a short distance, of the order of hundreds of micrometres [94, 95, 96]. Considering this, the potential treatment strategies of the gas plasma for skin cancer with the most promising impact can be broadly categorised as topical administration to superficial malignancies limited to the epidermis and postoperative treatment of the surgical bed.

### 4.1 Melanoma

Lentigo melanoma (LM) is a precursor to malignant melanoma and is an example of how topical application of cold medical gas plasma may play a significant role. Also known as Hutchinson's freckle, it is characterised by a superficial tumour where malignant cells are confined to the epidermis. The standard approach of surgery for LM can be fraught with difficulty, mainly when the lesion is located in sensitive anatomical areas or the patient's age and lesion size pose surgical risks [97]. Existing nonsurgical treatment alternatives such as radiotherapy, cryotherapy, laser ablation and topical immunomodulatory therapies, though less invasive and more cosmetically acceptable, can sometimes fall short in terms of controlled (i.e., spatially restricted) localised treatment of the problematic spots, typically with rates of 80%–90% compared to surgical excision [98]. Gas plasma delivered topically may be able to adequately penetrate the epidermis to treat LM either as monotherapy or in combination with other nonsurgical approaches to improve the outcomes of the localised spot treatment.

In cases of locally advanced or metastatic melanoma, systemic treatments such as BRAF and MEK inhibitors and immune checkpoint inhibitors (ICI) have transformed prognosis, with a significant portion of patients surviving at least 5 years post‐ICI treatment [99]. However, there remains a need for complementary treatment options, particularly for unresectable locoregional disease and the potential to de‐escalate the dosing of radiotherapy and chemotherapy. In this context, high‐intensity, targeted intratumoral therapy (HIT‐IT) has gained traction, maximising its concentration at the disease site and promoting both a localised and systemic immune response [100]. Talimogene laherparepvec (T‐VEC), the only treatment with regulatory approval for intratumoral administration in unresectable metastatic melanoma, exemplifies this approach [101]. In this context, the use of gas plasmas, in combination with ICI or other intratumoral agents, could offer a novel and effective approach to treating unresectable melanoma. A combination of plasma treatment of superficial tumours, ulcerating tumours or resection margins with immune checkpoint blockade using injectable antibodies such as anti‐CTLA4 and anti‐PD1 is reasonable. It might be an option for patients not benefiting from ICI monotherapy [102]. Primary ICI resistance due to a low tumour mutational burden (TMB) [103] might be overcome by creating tumour‐associated antigens (TAA) on‐site on the plasma‐treated tumour. Preclinical studies have shown elevated levels of effector T‐cells in plasma‐treated tumours, downregulation of PD1 and synergistic antitumor efficacy of ICI with plasma [35, 104]. The efficacy of ICI in nonmelanoma skin cancers (NMSCs) is currently under investigation and shows promising results [105]. Similar to melanoma, combining with plasma might increase ICI efficacy in NMSCs. Still, more in vivo preclinical studies or clinical studies proving the value of such combinations are currently lacking. Due to the poor penetration of the plasma‐produced reactive agents into skin and tissue, an intratumor delivery system in the form of liquid or hydrogel may be a valuable option. At the same time, gas plasma‐enhanced whole‐tumour vaccines seem an auspicious approach, as preclinical research suggests [35, 106].

### 4.2 Nonmelanoma Skin Cancers

The majority of nonmelanoma skin cancers (NMSCs), such as SCCs and BCCs, are resectable with clear margins using either a standard wide local excision or Moh's micrographic surgery [107]. However, some tumours have adverse risk factors such as aggressive histological subtype, large size or deep invasion, perineural invasion or close margins that are associated with local recurrence and warrant adjuvant treatments. Postoperative radiotherapy is commonly used in this setting but is associated with both acute and late toxicities such as radiation dermatitis, telangiectasis, fibrosis and lymphoedema [108]. Intraoperative delivery of gas plasma directly to the tumour bed and TME may address microscopic tumours to improve local control and may obviate the need for additional adjuvant therapy in intermediate‐risk cases or allow reduced doses of ionising radiation. This approach was recently investigated with some success in a Phase I clinical trial in patients with Stage IV or recurrent solid tumours who underwent surgical resection combined with intra‐operative gas plasma therapy [84].

Some patients develop multiple skin cancers in large areas of sun‐exposed skin in a disease process known as extensive skin field cancerisation (ESFC). The aetiology of this condition is chronic exposure to solar ultraviolet radiation, leading to the development of a field of mutant cells predisposed to forming neoplastic growth. The resulting skin lesion from this malignant growth can clinically represent a neoplastic continuum of conditions ranging from AKs through cSCC in situ (intraepidermal cancer; IEC) to invasive cSCC. ESFC represents a complex and challenging disease entity to treat with traditional treatment options [109]. Although a combination of field treatments [topical treatments, such as photodynamic therapy (PDT)] and lesion‐directed therapies has been recommended in international guidelines, the effectiveness is variable and the durability of control is limited. Gas plasma is emerging as a potential cancer therapy and an attractive adjuvant (e.g., after surgery) to existing cancer treatments [110]. The strategy has been explored in an early‐phase clinical trial of patients with AK, demonstrating a significant response of these lesions to the gas plasma exposure, which appeared to reduce the effects of chronic photodamage and subsequently induced lesions [74]. Unlike other therapies, there was no evidence of skin atrophy, and as gas plasma is already accepted as a clinical standard for treating nonhealing ulcers, there is no concern in treating high‐risk areas such as the lower limb extremities [111].

### 4.3 Practical Considerations for Clinical‐Grade Gas Plasma Equipment for Skin Cancer

Most gas plasma devices that have shown promising effects against skin cancer cells across various test models are of experimental origin, that is, they are usually sources only working in a laboratory environment, need expensive or bulky equipment and/or are not suitable or safe to be operated in the clinical setting. There are several exceptions [112], however, such as two exceptions are the microplaster devices as research versions of the clinically approved SteriPlas [113, 114] and the kINPen gas plasma jet [112]. In general, medical devices worldwide must demonstrate compliance with stringent requirements for safety and performance before obtaining marketing authorisation. While medical indications and treatment standards are universal, regulatory intricacies vary across different markets, often being country specific. Nevertheless, the primary objective shared by all regulatory systems is to facilitate patient access to high‐quality, safe and effective medical devices while preventing the distribution of unsafe products. To address the challenges posed by diverse national regulations, medical device institutions and authorities, the International Medical Device Regulators Forum (IMDRF), a group of medical device regulators from around the world (e.g., Australia, Brazil, Canada, China, Europe, Japan, South Korea and USA), has voluntarily collaborated to harmonise regulatory requirements for medical products across different countries. Such regulatory frameworks are subject to continuous revision to integrate the latest scientific findings and enhance the safety and performance of medical devices. In Europe, for instance, the recent update of the MDR guidelines stipulates that device‐specific clinical and preclinical data must support the benefits and safety of devices. The practice of cross‐referencing new gas plasma devices seeking approval with data from previously approved devices is no longer sufficient for obtaining marketing authorisation.

In addition, the field of plasma medicine sees a significant potential for including artificial intelligence (AI) and, specifically, machine learning (ML) processes in and for medical devices. A key advantage of AI/ML lies in its ability to derive valuable insights from the extensive data generated during healthcare delivery. Digital health technologies are playing an increasingly vital role in various aspects of health and daily life, with AI/ML driving significant progress in the field. Ensuring the safety and effectiveness of these innovative devices and enabling them to realise their full potential in assisting people are central to the public health missions of Medical Device Regulators. Interest in medical devices with AI/ML functionality has surged, particularly with the development of large language models (LLMs) [115, 116]. LLMs, such as ChatGPT, Llama, Claude and PaLM, are AI models trained on massive data sets, enabling them to recognise, summarise, translate, predict and generate content. The FDA has reviewed and authorised nearly 700 AI/ML‐enabled devices across various medical fields over the past decade, expecting this trend to persist. Radiology has seen the most consistent rise in submissions of AI/ML‐enabled devices among all specialities. Notably, as of October 19, 2023, the FDA has not authorised any device using generative AI, artificial general intelligence (AGI) or powered by large language models. In cancer research and treatment, AI‐based systems can enhance the accuracy and consistency of cancer diagnosis, reducing error rates for pathologists. Predictive AI models can estimate an individual's likelihood of developing cancer by identifying relevant risk factors. Additionally, integrating big data with AI allows medical experts to tailor customised treatments for cancer patients, potentially leading to less severe side effects than generalised therapies. In skin cancer therapy, AI‐assisted imaging analysis [117, 118] (e.g., for dermatoscopic images) could be helpful to decide whether plasma treatment would be beneficial, for example, based on the invasiveness of the lesion, as well as for follow‐up assessment. In addition, machine learning could be trained on real‐time monitoring data on optical emission spectroscopy, changes in the effective power of the device or sound, to learn when being close to tissue or not, potentially allowing future robotics to perform in some cases time‐consuming gas plasma treatment of patient skin, skin lesions or wounds. Moreover, AI could be used to assist with supervising the progress of the tissue based on photographs before and after treatment to allow calculating visual indicators that help the clinician with making decisions on whether to continue the gas plasma therapy or which treatment frequency would be necessary, provided data sets with necessary sizes would become available over the years.

Regarding device standards, gas plasmas derived from different plasma sources might differ in their physical and biological effects and potential risks posed for patients and therapists. Therefore, an explicit risk and performance assessment must be carried out to ensure that the use of plasma on humans is ‘safe’ and that the therapeutic window defined for the desired application is appropriate. For this assessment, detailed plasma diagnostics must be performed to identify the plasma components (especially UV dose, toxic gas emission and current through the target) and to determine their respective concentration or values for, for example, different plasma settings and treatment times, using various experimental and theoretical modelling tools. The results from these measurements should then be compared with existing published threshold values and interpreted accordingly. To establish general requirements for plasma sources in medicine, a DIN SPEC 91315 was published by Mann and colleagues in 2014 [119]. The goal of the document is to provide essential criteria for plasma sources to be used in medical applications. Furthermore, the efficacy of medical plasma sources and the safety of users (e.g., experimenters, patients and therapists) are of great importance. The tests described in the DIN SPEC 91315 are easy to adapt if standard laboratory equipment is available. Still, they must be adjusted to every plasma source concerning individual treatment conditions. It is widely acknowledged that treatment results obtained with one plasma source cannot be transferred to another plasma device. The reason for this nontransferability is the biological effects caused by the ‘cocktail’ of reactive species, radiation (above all ultraviolet light), the electrical current flow from plasma to the body, the flow of working gas and the heat transfer to the treated surface, depending on the plasma generation technology. As such, a precise characterisation of the to‐be‐applied gas and an indication‐specific device setting for standardisation should be mandatory.

## 5 Conclusion

Based on many auspicious preclinical data, we are witnessing promising momentum in efforts to include medical gas plasmas in skin cancer care. However, the time is ripe for clinical evidence to estimate the realistic benefit of this technology in dermato‐oncology therapy schemes. Several challenges and opportunities for research and development are needed for plasma‐based skin cancer treatment approaches to become widely adopted clinical realities. These opportunities appear prominently in the three key areas of plasma device standardisation, mechanisms of action of reactive agents produced by the plasmas and the development of standardised treatment modalities and protocols. Here, we will only highlight one typical opportunity from each area. First, the plasma devices must be designed to comply with the applicable safety and operational standards and achieve the desired therapeutic effects. For example, the temperature of the gas flow effluent from plasma jets should be effectively controlled by the end‐users so that they operate at a temperature close to the local temperature of the treated area of the skin or other tissues. Any electric shock effects on the patients during the treatment should be eliminated. Second, more profound insights into the specific mechanisms of action of reactive agents produced by the plasmas are needed to develop the power supplies, electrode configurations and gas feeds for the plasma devices to ensure the effectiveness of the plasma treatments. In this regard, a common approach based on determining the standard doses of skin exposure and other tissues to the plasma would be very beneficial for developing specific single or combinatorial treatment modalities and protocols. While this approach is common in some radiation‐based therapies, it is still not used in plasma medicine, primarily because of the overwhelming diversity and different mechanisms of action of the reactive agents (e.g., electrons, ions, ROS, RNS, other excited molecules and radicals, light, intrinsic electric fields and heat) produced by the plasmas. Third, a better understanding of the specific effects of these reactive agents needs to be translated into simple and user‐friendly details of the treatment processes to be applied in clinical practice. The optimal doses of plasma exposure need to be elaborated to achieve the desired bio‐medical effects, such as selective cancer cell apoptosis, without harming neighbouring healthy cells. Ideally, the doses required for such effects should be determined based on detailed studies of mechanisms of biological effects of gas plasmas in cancer biology research and without time‐ and effort‐consuming trial‐and‐error experimentations. Importantly, the properly determined and validated doses of plasma exposure should be converted into detailed practical treatment protocols, including plasma exposure times, intensities and repetition frequencies, safe distances between the plasma device (e.g., plasma jet nozzle or DBD electrode) and the treated skin surface, operating gases and environments (including room temperature and moisture) and no doubt other factors. Integrating AI‐based approaches for the above purposes is highly promising and should be considered soon. Practical implementation of these critical factors is required to ensure the successful translation of the currently mostly laboratory‐based plasma medicine research into clinical reality, which demands patient safety, treatment efficacy and regulatory compliance. Therefore, integrated and coordinated efforts of multidisciplinary researchers, engineers and clinical end‐users are vital for the widespread translation of plasma technologies into clinical skin cancer treatments.

## Author Contributions

S.B. designed the review; L.M., G.R., K.O. and S.B. wrote the draft; all authors wrote and reviewed the paper.

## Conflicts of Interest

K.R. is affiliated with Neoplas MED GmbH (Greifswald, Germany), which produces and distributes the kINPen MED argon plasma jet. All other authors declare that there is no Conflicts of Interest regarding the publication of this paper.

## Data Availability

Data sharing is not applicable to this article as no new data were created or analyzed in this study.
